# The Effects of Probiotic *Bacillus* Spores on Dexamethasone-Treated Rats

**DOI:** 10.3390/ijms242015111

**Published:** 2023-10-12

**Authors:** Andreea Ioana Inceu, Maria Adriana Neag, Adrian Catinean, Corina Ioana Bocsan, Cristian Ioan Craciun, Carmen Stanca Melincovici, Dana Maria Muntean, Mădălin Mihai Onofrei, Raluca Maria Pop, Anca Dana Buzoianu

**Affiliations:** 1 Department of Pharmacology, Toxicology and Clinical Pharmacology, Iuliu Hatieganu University of Medicine and Pharmacy, 400337 Cluj-Napoca, Romania; andreea.inceu.i@gmail.com (A.I.I.); corinabocsan@yahoo.com (C.I.B.); cristian.craciun@umfcluj.ro (C.I.C.); raluca_parlog@yahoo.com (R.M.P.); abuzoianu@umfcluj.ro (A.D.B.); 2 Department of Internal Medicine, Iuliu Hatieganu University of Medicine and Pharmacy, 400006 Cluj-Napoca, Romania; catinean@gmail.com; 3 Department of Histology, Iuliu Hatieganu University of Medicine and Pharmacy, 400349 Cluj-Napoca, Romania; carmen.melincovici@umfcluj.ro (C.S.M.); onofrei.madalin.mihai@elearn.umfcluj.ro (M.M.O.); 4 Department of Pharmaceutical Technology and Biopharmaceutics, Iuliu Hatieganu University of Medicine and Pharmacy, 400010 Cluj-Napoca, Romania; dana.muntean@umfcluj.ro

**Keywords:** *Bacillus* spores, dexamethasone, inflammation, hyperglycemia, dyslipidemia, oxidative stress

## Abstract

Glucocorticoids are effective anti-inflammatory and immunosuppressive agents. Long-term exposure is associated with multiple metabolic side effects. Spore-forming probiotic bacteria have shown modulatory properties regarding glycolipid metabolism and inflammation. The aim of this study was to evaluate, for the first time, the effects of *Bacillus* species spores (*B. licheniformis*, *B. indicus*, *B. subtilis*, *B. clausii*, and *B. coagulans*) alone and in combination with metformin against dexamethasone-induced systemic disturbances. A total of 30 rats were randomly divided into 5 groups: group 1 served as control (CONTROL), group 2 received dexamethasone (DEXA), group 3 received DEXA and MegaSporeBiotic (MSB), group 4 received DEXA and metformin (MET), and group 5 received DEXA, MSB, and MET. On the last day of the experiment, blood samples and liver tissue samples for histopathological examination were collected. We determined serum glucose, total cholesterol, triglycerides, tumor necrosis factor-alpha (TNF-α), interleukin-6 (IL-6), interleukin-10 (IL-10), catalase, total antioxidant capacity (TAC), and metformin concentration. DEXA administration caused hyperglycemia and hyperlipidemia, increased inflammation cytokines, and decreased antioxidant markers. Treatment with MSB reduced total cholesterol, suggesting that the administration of *Bacillus* spores-based probiotics to DEXA-treated rats could ameliorate metabolic parameters.

## 1. Introduction

In the context of the coronavirus disease 2019 (COVID-19) pandemic, glucocorticoid (GC) therapy in the form of DEXA has emerged as an effective measure against severe acute respiratory syndrome coronavirus 2 (SARS-CoV-2), due to its powerful anti-inflammatory and immunosuppressive effects. Described as ‘flight and fight’ hormones, the acute secretion of GCs allows energy adaptation to situations of danger, stress, or metabolic imbalance. However, long-term exposure to GCs is associated with multiple metabolic side effects, affecting the basal metabolism of carbohydrates and lipids [1].

GCs produce dysregulation of glucose-induced insulin release and insulin resistance by activating specific receptors that decrease the uptake of glucose in the peripheral tissues and increase the hepatic production of glucose [2,3]. Under acute stress conditions, GCs stimulate β-cell function and insulin secretion to supply the demand for glycemia. The long-standing activation of β-cells leads to insulin resistance and later to β-cell apoptosis, decreased insulin biosynthesis, and subsequently reduced insulin secretion [2]. DEXA can cause β-cell apoptosis via the activation of cellular oxidative stress and the generation of reactive oxygen species (ROS) [4]. Moreover, DEXA has been associated with changes in gut microbiota richness and diversity, suggesting additional mechanisms that are activated in the dysregulation of glycolipid metabolism [5]. Steroid-induced diabetes is responsible for 2% of the cases of diabetes mellitus in the general population [6]. Therapeutic measures depend on the duration and type of glucocorticoid therapy and include non-insulin glucose-lowering drugs and insulin-based agents [2].

Probiotics are live microorganisms that confer favorable properties for the host’s health via amelioration of gut microbiota composition and protection of the gut environment [7]. Probiotic supplements have been associated with beneficial effects regarding glycemic balance, lipid profile, inflammation indicators, and blood pressure values in patients with diabetes mellitus type 2 [8]. The observed anti-diabetic effects are due to the modulation of gut microbiota composition, regulation of immune reactions, and improvement of energy metabolism [9]. Spores-based probiotics have additional benefits, such as higher resistance to gastric acid and increased stability at room temperature [10]. MSB, a probiotic mixture of five spore-forming *Bacillus* strains, has enriched the microbial diversity in an in vitro model of a simulator of the human intestinal microbial ecosystem (SHIME^®^) and ameliorated the production of short-chain fatty acids [11]. Moreover, the administration of MSB decreased dietary endotoxemia, serum pro-inflammatory cytokines, and triglyceride levels in human subjects [12].

Traditionally, MET, a biguanide-derived drug, is used in the treatment of diabetes mellitus type 2 as a modulator of insulin sensitivity by decreasing hepatic gluconeogenesis and lipogenesis. Hepatic glucose production is regulated based on a multi-organ communication activated by MET that includes the gut, the blood, and the liver. In the intestines, MET stimulates gut metabolism and glucose utilization, increases the secretion of the incretins, and modifies the microbiota composition. In the blood, inflammation is suppressed by MET treatment. In the liver, MET impact’s mitochondrial function and modulates molecular pathways involved in glucose and lipid metabolism [13]. MET showed beneficial effects regarding glucose and lipid metabolism, liver markers, and inflammation in patients on systemic glucocorticoid therapy for inflammatory diseases [14].

Taking this into consideration, this study aimed to evaluate the effect of *Bacillus* spore probiotics, alone and in combination with MET, on metabolic, inflammatory, and oxidative disturbances induced by DEXA administration in rats.

## 2. Results

### 2.1. Biochemical Results

**Figure 1:**
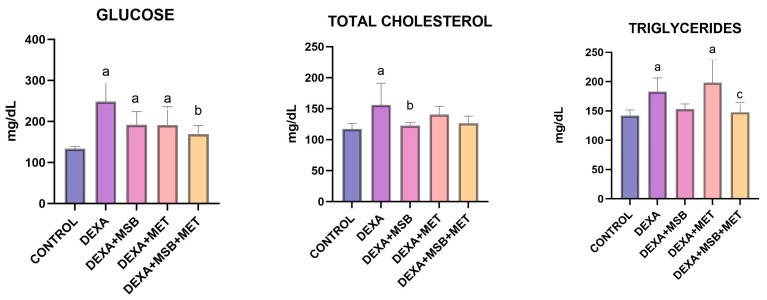
Serum glucose, total cholesterol, and triglycerides concentration. a: *p* < 0.05 compared to the CONTROL group; b: *p* < 0.05 compared to the DEXA group; c: *p* < 0.05 compared to the DEXA + MET group. Abbreviations: DEXA, dexamethasone; MSB, MegaSporeBiotic; MET, metformin. The bars represent mean values with a standard deviation.

Serum glucose, total cholesterol, and triglyceride levels are represented in Figure 1.

The serum concentration of glucose (mean ± SD) increased significantly after DEXA administration compared to the CONTROL group (248.60 ± 47.37 mg/dL vs. 133.50 ± 5.70 mg/dL, *p* < 0.0001). In DEXA-treated rats, treatment with MSB or MET lowered the values of serum glucose by 22% (191.80 ± 32.24 mg/dL) and 23% (191.50 ± 44.53 mg/dL); however, without statistically significant differences (*p =* 0.054 and *p* = 0.052, respectively). However, the coadministration of MSB and MET in DEXA-treated rats significantly reduced serum levels of glucose (169.50 ± 21.03 mg/dL, *p* = 0.003) compared to the DEXA group.

DEXA administration induced a significant increase in the total cholesterol serum levels (mean ± SD, 156.10 ± 35.10 mg/dL) versus the CONTROL group (117.30 ± 8.84 mg/dL, *p =* 0.008). The DEXA + MSB group showed a statistically significant difference in total cholesterol levels (122.70 ± 5.17 mg/dL) compared to the DEXA group (*p =* 0.02). MET administration or the combination of MET and MSB in DEXA-treated rats decreased the concentration of total cholesterol (140.50 ± 13.45 mg/dL and 126.30 ± 11.71 mg/dL, respectively) compared to the DEXA group, but without statistically significant results (*p =* 0.578 and *p =* 0.061, respectively).

Compared to the CONTROL group, serum triglycerides were significantly higher in the DEXA-treated rats (mean ± SD, 141.90 ± 9.90 mg/dL versus 183 ± 23.14 mg/dL, *p =* 0.03). In the DEXA + MSB and DEXA + MSB + MET groups, triglyceride levels decreased by 16.4% (153 ± 8.85 mg/dL) and 19.23% (147.80 ± 16.28 mg/dL), respectively, compared to the DEXA group; however, the differences were not statistically significant (*p =* 0.174 and *p =* 0.08, respectively). MET administration did not significantly alter the triglycerides serum concentration in DEXA-treated rats compared to the DEXA group (198.20 ± 39.41 mg/dL). In the DEXA + MSB + MET group, serum triglyceride levels were significantly reduced versus the DEXA + MET group (*p =* 0.005).

### 2.2. TNF-α, IL-6 and IL-10 Levels

**Figure 2:**
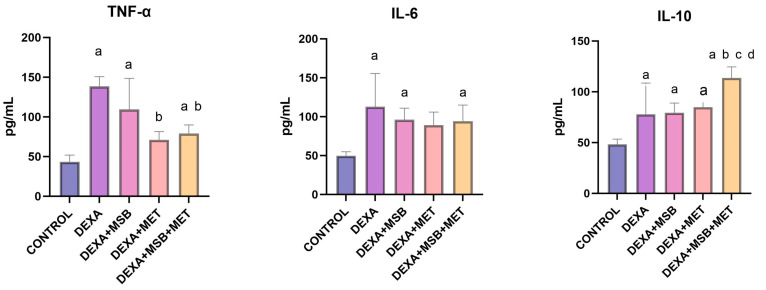
Serum TNF-α, IL-6, and IL-10 levels. a: *p* < 0.05 compared to the CONTROL group; b: *p* < 0.05 compared to the DEXA group; c: *p* < 0.05 compared to the DEXA + MET group; d: *p* < 0.05 compared to the DEXA + MSB group. Abbreviations: TNF-α, tumor necrosis factor alpha; IL, interleukin; DEXA, dexamethasone; MSB, MegaSporeBiotic; MET, metformin. The bars represent mean values with a standard deviation.

Serum TNF-α, IL-6, and IL-10 concentrations are shown in Figure 2. The serum levels of TNF-α (mean ± SD) in healthy CONTROL rats were 43.60 ± 8.26 pg/mL. In the DEXA-treated rats, serum TNF-α concentration was significantly increased compared to the healthy CONTROL group (138.60 ± 12.26 pg/mL, *p* < 0.0001). Treatment with MSB reduced the serum levels of TNF-α by 21% (109.40 ± 39.28 pg/mL); however, the decrease was not statistically significant (*p* = 0.11). However, MET treatment (DEXA + MET group) and the combination of MSB and MET (DEXA + MSB + MET group) significantly reduced the serum concentration of TNF-α compared to the DEXA group (71.14 ± 10.44 pg/mL; *p* < 0.0001 and 79.08 ± 10.92 pg/mL; *p* = 0.0002, respectively).

The serum IL-6 concentration (mean ± SD) was significantly increased in the DEXA-treated rats (112.90 ± 42.65 pg/mL; *p* = 0.0009) compared to the CONTROL group (49.96 ± 4.95 pg/mL). In the DEXA + MSB group, MSB administration reduced serum IL-6 levels by 14.8% (96.13 ± 14.85 pg/mL); however, the decrease was not statistically significant (*p* = 0.735). DEXA + MET and DEXA + MSB + MET groups exhibited reduced levels of IL-6 (89.01 ± 16.94 pg/mL and 94.22 ± 20.76 pg/mL, respectively), but without statistically significant differences (*p* = 0.422 and *p* = 0.652, respectively).

The serum IL-10 levels (mean ± SD) significantly increased in the DEXA-treated group (77.98 ± 30.76 pg/mL, *p* = 0.021) compared to the CONTROL group (48.30 ± 5.10 pg/mL). In the DEXA + MSB + MET group, serum IL-10 concentration was significantly higher (113.70 ± 10.88 g/mL, *p* = 0.004) than in the DEXA group. The association between DEXA, MSB, and MET also significantly increased IL-10 levels compared to the DEXA + MSB group (79.43 ± 9.48 pg/mL, *p =* 0.006) and the DEXA + MET group (84.87 ± 5.20 pg/mL, *p =* 0.02), respectively.

### 2.3. TAC and Catalase

**Figure 3:**
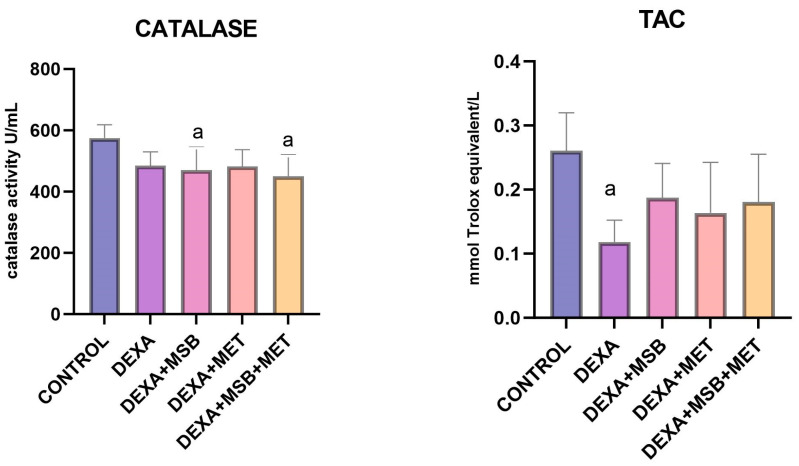
TAC and catalase levels. a: *p* < 0.05 compared to the CONTROL group; Abbreviations: TAC, total antioxidant capacity; DEXA, dexamethasone; MSB, MegaSporeBiotic; MET, metformin. The bars represent mean values with a standard deviation.

Serum levels of TAC and catalase are shown in Figure 3.

The catalase activity (mean ± SD) in CONTROL-healthy rats was 575.30 ± 43.16 U/mL. In the DEXA-treated rats, catalase activity decreased by 15.87% (484.0 ± 45.89 U/mL) without a statistically significant difference (*p* = 0.089). Administration of MSB, MET, or the combination of both MSB and MET slightly reduced the catalase activity (469.80 ± 76.13 U/mL, 482.6 ± 54.33 U/mL, and 450.10 ± 70.30 U/mL, respectively).

**Figure 4:**
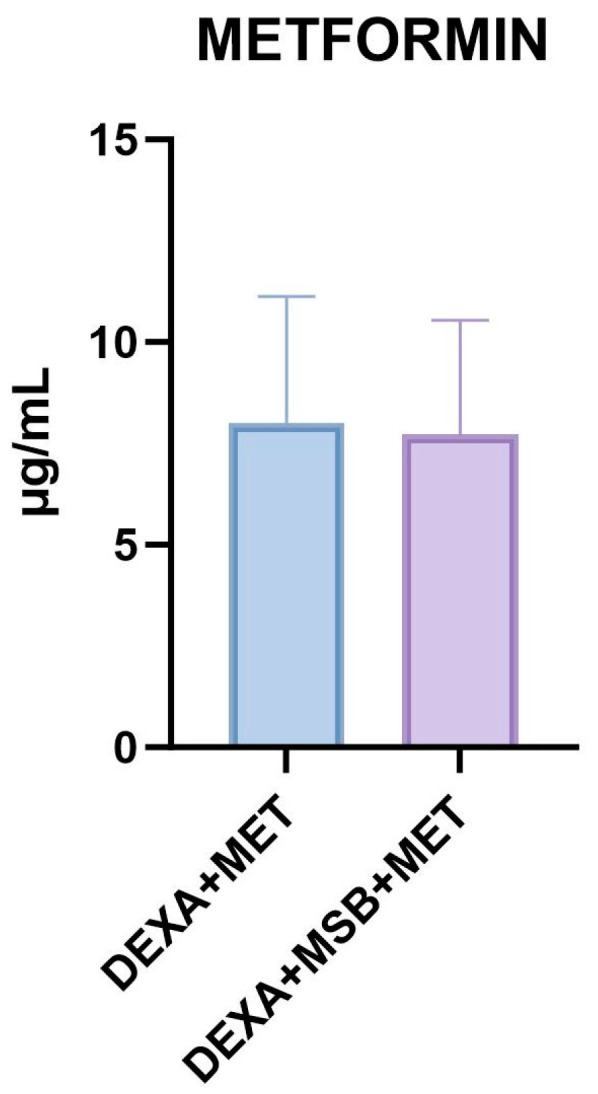
Serum metformin levels. Abbreviations: DEXA, dexamethasone; MSB, MegaSporeBiotic; MET, metformin. The bars represent mean values with a standard deviation.

In the DEXA group, TAC (mean ± SD) was significantly reduced (0.118 ± 0.033, *p* = 0.004) compared to the CONTROL group (0.260 ± 0.059). As shown in Figure 4, a higher TAC was observed in all treated groups compared to the DEXA group, without statistically significant differences.

### 2.4. Metformin

The metformin serum concentration is shown in Figure 4. In the DEXA + MSB + MET group, the administration of MSB did not alter the serum concentration of MET compared to the DEXA + MET group (8.01 ± 3.12 µg/mL versus 7.72 ± 2.81 µg/mL).

### 2.5. Histopathology

Histological analysis of liver tissue sections was focused on assessing the changes in the hepatic lobular architecture, hepatocyte degeneration, lipid accumulation in hepatocytes, inflammation, and blood vessel congestion, as well as restoration of the hepatocyte structure and liver architecture.

**Figure 5:**
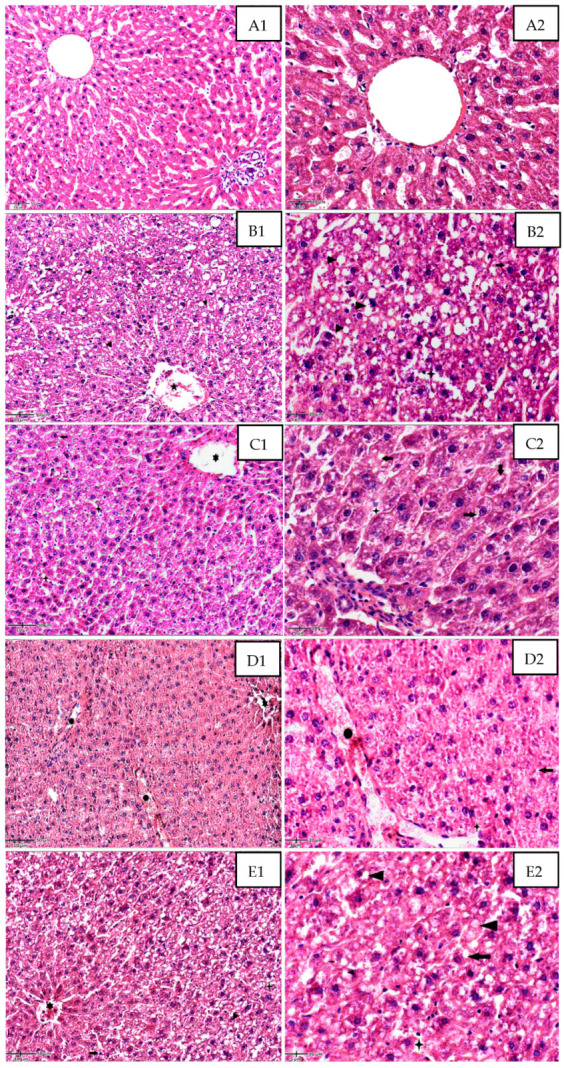
Photomicrographs of liver sections stained by H&E. (**A**): CONTROL group; ((**A1**), 20× and ((**A2**), 40×). (**B**): DEXA-treated rat group; ((**B1**), 20×); and ((**B2**), 40×). Alterations of hepatocytes structure and hepatic lobule architecture; enlarged, ballooned cells, with intracytoplasmic small lipid droplets in the perinuclear area(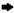); large lipid droplets within the hepatocytes (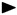); degenerated eosinophilic hepatocytes with pyknotic and hyperchromatic nuclei (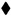); loss of cellular borders; vascular congestion in the central vein (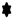); moderately dilated sinusoids with mild inflammatory infiltrate (polymorphonuclears neutrophils) within the sinusoids (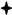). (**C**): DEXA + MSB treated rat group; ((**C1**), 20×) and ((**C2**), 40×). Partial reduction in lipid accumulation with small perinuclear lipid droplets within the hepatocytes (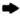); moderate improvement in hepatocytes structure; mild central vein ectasia (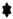); sinusoidal stasis and dilatation with few inflammatory cells (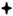). (**D**)**:** DEXA + MET-treated rat group; ((**D1**), 20×); and ((**D2**), 40×). Important restoration of the hepatic architecture and hepatocyte structure, with a significant reduction of intra-hepatocyte lipid content (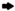); congestion within the portal vessels (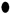) and sinusoids (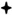); mild central vein congestion and stasis (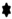). (**E**): DEXA + MSB + MET-treated rat group; ((**E1**), 20×); and ((**E2**), 40×). Poor improvement in hepatic lobular architecture and hepatocyte structure with still disorganized cords of cells and high intracellular lipid accumulation (intra-hepatocyte small (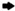) and large lipid droplets) (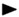); central vein congestion with intraluminal lymphocytes; (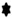); moderate inflammatory infiltrate within the dilated sinusoids (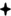). Abbreviations: H&E, hematoxylin, and eosin.

The CONTROL group exhibited a normal architecture of the classic hepatic lobules, with linear cords of hepatocytes separated by sinusoids, arranged around the central vein (Figure 5(A1,A2)).

DEXA-treated rats (Figure 5(B1,B2)) showed alterations in hepatocyte structure and hepatic lobule architecture. Mild hepatocyte hypertrophy was detected, with enlarged, ballooned cells having a pale-staining cytoplasm and enlarged nucleus, as well as intracytoplasmic lipid accumulation with small lipid droplets in the perinuclear area and large lipid droplets that occupy less than half of the cell. Moreover, some degenerated eosinophilic hepatocytes with pyknotic, hyperchromatic nuclei and loss of cellular borders may be identified, along with vascular congestion in the central vein and dilated sinusoids. A moderate inflammatory infiltrate can also be seen within the sinusoids and portal space, with lymphocytes and a few polymorphonuclear neutrophils.

MSB and DEXA administration provide a partial reduction in lipid accumulation with small perinuclear lipid droplets within the hepatocytes and moderate improvement in hepatocyte structure. Mild central vein ectasia as well as stasis and occasional inflammatory cells within the dilated sinusoids may be seen (Figure 5(C1,C2).

A more important histologic restoration of the hepatic architecture and hepatocyte structure was achieved after MET and DEXA administration (Figure 5(D1,D2)), with an important reduction of intra-hepatocyte lipid content. Congestion within the portal vessels and sinusoids, along with mild central vein congestion, are observed.

Treatment with MET, MSB, and Dexa (Figure 5(E1,E2)) shows poor improvements in hepatic lobule architecture and hepatocyte structure, with still disorganized cords of cells and high intracellular lipid accumulation, with the cells displaying small and large lipid droplets that occupy less than half of the cell. Central vein congestion with intraluminal lymphocytes, as well as dilated sinusoids with moderate inflammatory infiltrate within the lumen, are identified on the sections.

## 3. Discussion

This present study focuses on the role of *Bacillus* spores-based probiotics, alone and in combination with MET, in DEXA-induced metabolic disorders in rats. Compared to the CONTROL group, DEXA generated hyperglycemia, hyperlipidemia, a pro-inflammatory and pro-oxidant systemic status, and morphological alterations similar to non-alcoholic fatty liver disease (NAFLD) [15].

GCs are frequently used drugs due to their anti-inflammatory and immunosuppressive properties, yet their side effects may limit their clinical benefit. DEXA, a powerful agent that has been proved to be effective in severe forms of SARS-CoV-2, is a long-acting glucocorticoid agent [1]. It increases the risk of hyperglycemia and diabetes mellitus and is often used for the development of insulin resistance in animal models. In the present study, DEXA administration, even for a relatively short period, induced hyperglycemia and hyperlipidemia, results similar to previous studies [16,17,18]. The development of steroids-induced diabetes is based on the binding of GCs to their specific receptors that activate the hepatic production of glucose, inhibit the peripheric use of glucose, stimulate lipolysis in adipose tissue by activating hormone-sensitive lipase, and impair the pancreatic secretion of insulin, leading to insulin resistance [2]. Moreover, GCs increase serum cholesterol and triglycerides by inhibiting the lipoprotein lipase, triggering de novo fatty acid synthesis, and increasing the secretion of very-low-density lipoprotein (VLDL) cholesterol [19,20].

In the current study, MSB decreased serum total cholesterol in DEXA-treated rats. This is the first study that presents the effects of *Bacillus* spores-based probiotics on DEXA-induced metabolic disturbances. DEXA exposure has shown alterations in the gut microbiota composition, together with accentuated fat deposition, circadian rhythm disorders, and abnormal metabolic parameters [5]. Gut dysbiosis and increased permeability of the gut barrier have been involved in the development of metabolic syndrome and associated systemic complications [21]. Thus, GCs-induced changes in the microbiota environment could contribute to the observed metabolic disturbances, suggesting a potential therapeutic role for the MSB. In both animal models of experimentally-induced diabetes and patients with diabetes mellitus type 2, probiotics demonstrated beneficial anti-diabetic properties regarding the improvement of gut barrier function with decreased permeability and consecutive endotoxemia, stimulation of incretin secretion, increase of insulin sensitivity and amelioration of glucose and lipid profile, immunomodulatory function, maintaining the balance between pro-inflammatory and anti-inflammatory cytokines, and oxidative stress regulation [9]. Probiotics containing *Bacillus subtilis* have been shown to regulate insulin and HbA1c levels and ameliorate glucose tolerance and lipid profiles in an animal model of streptozotocin-induced diabetes [22]. In high-fat diet animals, probiotics containing *Bacillus licheniformis* or *Bacillus coagulans* significantly improved serum cholesterol and triglycerides [23,24]. The observed results of *Bacillus* spores-based probiotics on DEXA-induced metabolic disturbances are similar to the effects of *Bacillus*-based probiotics on metabolic disorders induced by different mechanisms, such as the high-fat diet, regarding glycolipid profile, inflammation biomarkers, and histological alterations of the hepatic tissue, suggesting that probiotics could activate some common mechanisms of action that influence metabolic pathways [25].

MET is a widely used drug for the treatment of diabetes mellitus type 2 and its complications. The main mechanism of action relies on the inhibition of the hepatic production of glucose and the improvement of insulin sensitivity; however, supplementary actions on metabolism, gut, and inflammation have been described [13]. In the DEXA+ MET group, MET administration reduced the serum concentration of glucose and total cholesterol; however, without observed effects on the serum triglycerides; the differences were not statistically significant. The effect of lowering total cholesterol and glycemia is similar to that noticed in previous studies of both patients receiving systemic glucocorticoid therapy and DEXA-treated rats [14,26]. The effect of MET on the non-significant alteration of triglycerides serum levels in the DEXA + MET group is in accordance with a systematic review that evaluated the effect of MET versus other antidiabetic drugs regarding lipid metabolism in patients with type 2 diabetes, showing that the decrease of the triglycerides induced by MET depends on the glucose-lowering effect, the doses of MET, and the duration of the treatment [27]. The non-significant change in the serum concentration of triglycerides noticed in this study could be explained by the short duration and low dose of MET. The amelioration of glycolipid metabolic parameters in the DEXA + MSB + MET group could be explained by the similar favorable effects of both MSB and MET [12,27].

DEXA, a known anti-inflammatory and immunosuppressive agent, increased the serum concentration of TNF-α and IL-6 in this study. GCs have been reported to increase pro-inflammatory gene expression (TNF-α, IL-6), depending on the time and dose of the exposure [28]. TNF-α, a pro-inflammatory cytokine, is also being secreted from adipocytes in insulin-resistant conditions [29]. TNF-α has been recognized as a mediator of insulin resistance due to the observed actions of decreasing the activity of the tyrosine kinase of the insulin receptor by promoting the serine phosphorylation of insulin receptor substrate 1 (IRS-I) [30] and reducing the expression of glucose transporter 4 (GLUT 4) in the adipose tissue [31]. IL-6 manifested dual properties, according to the tissue and metabolic environment. In the liver and adipose tissue, IL-6 induces insulin resistance due to insulin receptor inhibition and enhances inflammation [32]. The observed results in this study regarding the serum TNF-α and IL-6 levels could be explained by the metabolic disturbances induced by DEXA that led to an insulin resistance state; this was observed in similar animal studies [33,34]. Furthermore, GCs-induced gut dysbiosis promotes the development of a pro-inflammatory state in the host that is associated with the development of metabolic disorders such as diabetes mellitus type 2 [5,35]. In the DEXA+ MSB rats, MSB lowered the serum concentration of TNF-α and IL-6, although the differences were not statistically significant, suggesting an anti-inflammatory effect. The short duration of treatment with MSB could explain the lack of statistically significant results. *Bacillus* spores-based probiotics displayed the ability to decrease systemic inflammation in human subjects [12] and in animal models of experimentally induced ulcerative colitis and acetaminophen-induced acute liver injury [36,37]. DEXA+ MET association reduced the inflammation profile of TNF-α and IL-6, results explained by the anti-inflammatory role of MET in diabetes mellitus [13,38]. In both animal models and human subjects with DEXA-induced metabolic disturbances, MET successfully alleviated inflammation [14,33].

IL-10 is mainly known as an inflammatory regulator that limits the production of pro-inflammatory molecules such as TNF-α, IFN-γ, IL-1, IL-2, and IL-6 and controls the activation of T cells via a direct effect on monocytes and macrophages [39]. DEXA-treated rats exhibited increased serum levels of IL-10. Previous studies have reported conflicting results regarding DEXA action over IL-10 secretion [40]. DEXA induced a biphasic alteration in lipopolysaccharides (LPS)-induced IL-10 secretion in blood cell culture, represented by up-regulation at lower doses and down-regulation at higher doses. In addition, IL-10 was shown to increase the concentration of glucocorticoid receptors and enhance DEXA activity [41]. Considering the serum pro-inflammatory state characterized by increased TNF-α and IL-6 induced by DEXA, increased IL-10 secretion could be explained by the direct effect of DEXA as an anti-inflammatory reaction [42]. MSB administration slightly increased the IL-10 levels compared to the DEXA-treated rats. This observed trend was also noticed in other animal models of high-fat diet or streptozotocin-induced diabetes, where probiotic *Lactobacillus plantarum* increased the expression of IL-10 while decreasing the expression of TNF-α and IL-6, providing immunomodulatory characteristics of probiotics [43]. In the DEXA + MET group, IL-10 serum levels were higher than in the DEXA group. In NAFLD models, MET treatment has been shown to manifest protective effects against the development of NAFLD in association with the improvement of inflammation markers such as IL-10, gut microbiota composition, and gut barrier integrity [44,45].

Free radicals can induce varying degrees of cellular damage related to the function of the intracellular defense system. Catalase is an enzyme with antioxidant activity, capable of maintaining redox homeostasis in the cell and influencing lipid peroxidation and the formation of hydroxyl radicals [46,47]. In our study, the level of plasma catalase decreased slightly in the DEXA group. This result is similar to that obtained by other researchers and is in accordance with the observed results for TAC, suggesting that the damage induced by DEXA is due to excess ROS and the consecutive depletion of the antioxidant system [48,49,50]. Moreover, in this study, we observed that catalase level was not significantly influenced by either MET, MSB, or their combination, even though there are studies of MET increasing catalase activity in a dose-dependent manner [51]. The noted results can be explained using a single dose of MET and the limited duration of the experiment. Regarding the *Bacillus* probiotic, similar results were obtained in a study using *Bacillus subtilis*, where this probiotic had no significant impact on catalase [52].

Moreover, DEXA reduced TAC compared to the CONTROL group, with results similar to previous studies [53]. ROS and oxidative stress are involved in the pathophysiology of DEXA-induced insulin resistance [54]. At the pancreatic level, hyperglycemia and glucotoxicity of β cells produce lipid peroxidation and diminish the antioxidant glutathione [17]. ROS induces dysfunction in β cells due to their decreased antioxidant capacity. Oxidative stress activation leads to apoptosis, impaired mitochondrial functionality, and alteration of the K ATP channels [55]. MSB increased the TAC in the DEXA-treated group, although the difference was not statistically significant. MSB showed antioxidant features observed in animal models of ulcerative colitis or acetaminophen-induced acute liver injury [36,37]. *Bacillus cereus* and *Bacillus coagulans* produce extracellular polysaccharides (EPS) that remove the ROS within the intestine, displaying antioxidant properties and protecting against oxidative stress-induced DNA damage [56,57]. Another antioxidant mechanism exhibited by *Bacillus* species is the synthesis of riboflavin and carotenoids, known antioxidant molecules [58]. MET administration increased TAC in DEXA+ MET rats versus DEXA-only treated rats. In animal models of DEXA-induced insulin resistance, MET demonstrated the properties of oxidative stress regulators, increasing the antioxidant marker glutathione [59]. The antioxidant mechanisms activated by MET are the upregulation of the activity of antioxidant enzymes, the trapping of hydroxyl radicals, and the inhibition of NADPH oxidase, the main enzyme that supplies intracellular ROS [60].

At the histological level, DEXA-treated rats displayed disruption of hepatocyte architecture, intracellular lipid accumulation, cellular injury, and inflammatory infiltrate, indicating NAFLD. These histological characteristics are similar to those observed in other studies of DEXA-treated rats [18,61]. GCs-induced NAFLD is produced via different molecular mechanisms versus other animal models of NAFLD that are diet-induced or leptin receptor-deficient mice [62]. The activation of glucocorticoid receptors in the liver and adipose tissue promotes the lipid buildup within the hepatocytes via the induction of different enzymes responsible for lipogenesis and lipid mobilization, whereas hyperinsulinemia and insulin resistance uphold these mechanisms [15]. The histological examination of the liver tissue after MSB administration in DEXA-treated rats displayed reduced lipid deposition and a mild tendency to improve hepatic structure. MSB has shown protective properties regarding hepatocyte necrosis and hepatic inflammation in an animal model of acetaminophen-induced acute liver injury [37]. Moreover, a probiotic mixture consisting of five *Bacillus* spp. alleviated hepatic steatosis induced by a high-fat diet, attenuated chronic inflammation in the liver, ameliorated insulin sensitivity, and improved the function of the gut barrier [25]. MET administration improved the histological characteristics of the DEXA-induced NAFLD, with results similar to those observed in previous research. In nondiabetic patients with NAFLD, MET administration for 12 months ameliorated liver fibrosis, necrosis, inflammation, and lipid deposition, normalized the serum liver profile, and enhanced insulin sensitivity [63]. In the DEXA + MSB + MET group, a similar histological aspect to the DEXA group was associated with increased IL-10 serum concentration [64]. We first hypothesized that a pharmacokinetic interaction regarding MSB administration could mediate the observed result; however, the serum concentration of MET was not different between these two groups. MSB and MET associations improved serum metabolic and antioxidant parameters due to the combination of mechanisms; however, they could impact the inflammation equilibrium in a more complex way. The reduced expression of TNF-α and IL-6 and the raised level of IL-10 could indicate a dysregulated inflammatory balance related to the histological disturbances. IL-10 expression becomes activated very early in the course of acute hepatic injury; however, it manifests a time-dependent effect on the control of inflammation in liver damage. In experimentally induced acute liver injury produced by carbon tetrachloride (CCl4), IL-10 secretion was activated 6 h after the initial injury and increased through the progression of hepatic histological damage without being correlated with histological amelioration. Although IL-10 regulates the pro-inflammatory cytokines, it does not affect the histological pattern of inflammation observed initially after hepatic injury. In animal models of hepatic fibrosis caused by long exposure to CCl4, IL-10 demonstrated anti-fibrotic effects, suggesting that IL-10 action is time-dependent and may involve a direct antifibrotic mechanism [65]. Moreover, in patients with chronic C hepatitis, circulating IL-10 levels were associated with sustained necroinflammation activity, suggesting a relationship between IL-10 and liver histology severity [66].

## 4. Materials and Methods

### 4.1. Agents and Chemicals

We used MSB probiotic capsules (Microbiome Labs, Saint Augustine, FL, USA), Metformin (MET) (Siofor, 1000 mg/tablet, Berlin- Chemie Ag, Berlin, Germany), and Dexamethasone Sodium Phosphate (DEXA) (Dexamethasone Phosphate Krka 4 mg/mL, Krka d.d. Novo Mesto, Novo Mesto, Slovenia), which are standard chemical compounds. All products were purchased from a public pharmacy. MSB, a probiotic blend of 4 × 10^9^ CFU from five gram-positive, spore-forming *Bacillus* species *(B. licheniformis*, *B. indicus*, *B. subtilis*, *B. clausii*, and *B. coagulans*), and MET were administered orally as a suspension in 1 mL of 1% carboxymethylcellulose (CMC, vehicle). DEXA was administered by an intraperitoneal (i.p.) injection.

### 4.2. Animals

Charles River Wistar albino male rats (*n* = 30) weighing between 220 and 270 g were obtained from the Center for Experimental Medicine and Practical Skills of Iuliu Hatieganu University of Medicine and Pharmacy. The animals were fed rat chow ad libitum and had free access to tap water. The rats were maintained under standard conditions of temperature (22 ± 2 °C), light (12 h light/dark cycles), and humidity. The rats were acclimated to these conditions for two days before starting the experiment.

The working animal protocol was revised and approved by the Ethics Committee of Iuliu Hatieganu University of Medicine and Pharmacy (no. AVZ262/15.09.2022) and by the National Sanitary Veterinary and Food Safety Authority (no. 336/14.10.2022). Specific regulations and amendments used in this study were from the “Guiding Principles in the Use of Animals in Toxicology” adopted by the Society of Toxicology (Reston, VA, USA) and all national laws regarding the protection of animals used for scientific research.

### 4.3. Experimental Design

**Figure 6:**
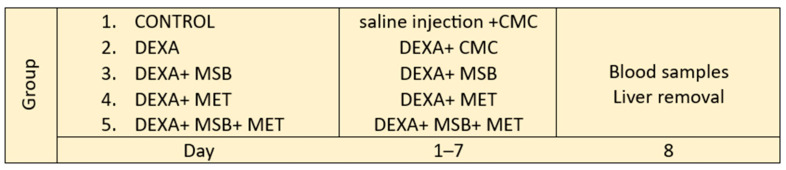
Experimental study design. Abbreviations: DEXA, dexamethasone; MSB, MegaSporeBiotic; MET, metformin; CMC, carboxymethylcellulose.

A total of 30 Wistar rats were divided into 5 groups, with 6 rats per group. Group 1 (CONTROL) served as the negative control and received the vehicle 1% CMC (7 days) and saline injection i.p. (7 days); group 2 (DEXA) received DEXA 1 mg/kg bw/day i.p. and the vehicle 1% CMC for 7 days and served as the positive control; group 3 (DEXA + MSB) received MSB (1 × 10^9^ colony forming units (CFU)/day/animal, 7 days); group 4 (DEXA + MET) received MET (100 mg/kg bw/day, 7 days); group 5 (DEXA + MSB + MET) received MSB (1 × 10^9^ CFU/day/animal, 7 days) and MET (100 mg/kg bw/day, 7 days); additionally, groups 3, 4, and 5 also received DEXA 1 mg/kg bw/day i.p. for 7 days. All treatments except for DEXA were administered orally through a feeding tube for 7 days. The recommended dose for humans is two capsules/day (2 × 4 × 10^9^ CFU/day); although the doses administered in humans and animals are different due to the accelerated metabolism in rodents, these doses showed beneficial effects regarding metabolic activity and modulation of the gut microbial community. Moreover, the same dose of MSB was used in similar experimental animal studies [11,12,36,67]. On day 8, blood samples and liver tissue samples were collected for further analysis. Blood was collected from the retro-orbital sinus plexus (periorbital) under anesthesia. The blood was allowed to coagulate, then the serum was separated by centrifugation at 4000 rpm for 15 min; the serum was stored at −20 °C for further biochemical analysis. Animals were sacrificed for a xylazine/ketamine overdose. The liver tissue was removed and preserved in 10% formaldehyde, dehydrated in graduated ethanol, and embedded in paraffin wax. The experimental design is shown in Figure 6.

### 4.4. Evaluation of Inflammatory and Biochemical Markers

TNF-α, IL-6, and IL-10 (serum levels of the proinflammatory cytokines) were quantified by enzyme-linked immunosorbent assay (ELISA) using commercially available ELISA kits (Rat TNF-α Standard TMB ELISA Development Kit, Rat IL-6 Standard ABTS ELISA Development Kit; PeproTech Inc., Rocky Hill, NJ, USA; Rat IL-10 Elabscience, China). The results were expressed as pg/mL. Biochemical parameters (serum glucose, total cholesterol, and triglycerides) were measured by an automatic biochemical analyzer according to the manufacturer’s protocols; the results were expressed in mg/dL.

### 4.5. Assessment of Oxidative Stress

TAC was assessed using a validated method previously described by Erel [68]. This technique measures the antioxidants’ capacity to decolorize the 2,2′-azinobis-3-ethylbenzothiazoline-6-sulfonate (ABTS^+^), which is a blue-green species, proportional to their concentration and antioxidant properties. ABTS^+^ is produced by the incubation of 2,2V-azinobis (3-ethylbenzothiazoline-6-sulfonate) (ABTS) with hydrogen peroxide in an acidic medium (acetate buffer: 30 mmol/L, pH 3.6). In this environment, concentrated ABTS^+^ molecules have higher stability over time. In conditions with a high pH acetate buffer (0.4 mol/L, pH 5.8), by adding a more concentrated acetate buffer solution, the deep-green color of concentrated ABTS^+^ molecules are slowly bleaching. This reaction of bleaching is enhanced in the presence of antioxidants proportional to their concentration and can be measured by using a spectrophotometer at 660 nm; the TAC of the sample is inversely related to the rate of bleaching. For the calibration curve, we used Trolox, a water-soluble analog of vitamin E; results were expressed as mmol Trolox equivalent/L.

For the measurement of catalase activity, a UV-spectrophotometric method was used, as previously described by Aebi [69]. The UV spectrophotometric method (Specord 250 Plus, Analytik Jena) monitors the modifications of 240 nm absorbance in a solution with a high concentration of hydrogen peroxide (10 µmole/mL) dissolved in 50 mM phosphate buffer at pH = 7.

### 4.6. Metformin Concentration

#### 4.6.1. Chromatography Apparatus and Conditions

A high-performance liquid chromatographic system (Agilent 1100 Series, Agilent Technologies, Santa Clara, CA, USA) composed of a binary pump, autosampler, column thermostat, and UV detector was used for MET determination. The chromatographic conditions were optimized by different means (different mobile phase combinations, using different columns) to obtain the best sensitivity for the analyte. Optimum separation conditions were obtained with a Gemini NX C18 150 × 4.6 mm, 5 µm column, with a mobile phase consisting of 10 mM phosphate buffer and methanol (60:40 (*v*/*v*)) in gradient mode, with column oven temperature maintained at 30 °C and elution monitored by UV detector at 233 nm.

#### 4.6.2. Sample Preparation

Protein precipitation was the preferred choice of separation because of the minimal steps involved in the extraction of the drug from the matrix. Approximately 0.1 mL of plasma and 0.3 mL of methanol were subjected to vortexing for about 1 min, followed by centrifugation for 5 min at 10,000 rpm. A total of 10 µL of supernatant was injected into the HPLC system.

The calibration curve proved to be linear between 10 and 80 µg/mL.

### 4.7. Histological Assessment

The excised liver tissue samples were fixed in a 10% buffered formalin solution for 24 h, dehydrated in ascending concentrations of alcohol solution, and embedded into paraffin wax. Afterward, the 4-5 µm liver sections were deparaffinized, rehydrated using a graded ethanol solution (100%, 90%, and 80%), and stained with routine hematoxylin-eosin (H&E) stain. The histological evaluation was performed with a Leica DM750 microscope, and the image caption was provided by a Leica ICC 50 HD camera connected to the microscope.

### 4.8. Statistical Analyses

All data were presented as mean ± standard deviation (SD). Firstly, the distribution of data were tested using the Shapiro-Wilk test and QQ plot representation. Once the normal distribution was assessed, the overall comparison between different groups was tested by using one-way analysis of variance (ANOVA), followed by comparisons between the pairs of groups by using post-hoc Tukey correction. To compare quantitative data from two independent groups with a normal distribution, the unpaired *t*-test was used. A *p*-value less than 0.05 was considered statistically significant. All statistical analyses were performed using GraphPad Prism, version 10 (GraphPad Software, Boston, MA, USA).

## 5. Conclusions

In conclusion, this study showed that MSB, a *Bacillus* spores-based probiotic, could impact the metabolic, inflammatory, histological, and oxidative stress disturbances induced by DEXA, with effects comparable to those of MET, a standard treatment for diabetes mellitus type 2. The obtained results reflect a crosstalk between metabolism and gut microbiota. The administration of these agents at the start of the DEXA exposure suggests that these supplements can partially prevent the structural and functional disorders that characterize steroid-induced diabetes. However, supplementary studies with a longer duration of treatment are necessary to support the use of probiotics as a preventive therapy for DEXA-treated patients.

## Data Availability

All the data are found in the present manuscript.
